# Gender Differences in Risk Factors of Congenital Hypothyroidism: An Interaction Hypothesis Examination

**DOI:** 10.5812/ijem.13946

**Published:** 2014-04-01

**Authors:** Shahab Rezaeian, Abbas Moghimbeigi, Nader Esmailnasab

**Affiliations:** 1 Department of Epidemiology, School of Health, Shiraz University of Medical Sciences, Shiraz, IR Iran; 2 Research Center for Health Sciences, Department of Epidemiology and Biostatistics, School of Public Health, Hamadan University of Medical Sciences, Hamadan, IR Iran; 3 Kurdistan Research Center for Social Determinants of Health (KRCSDH), School of Medicine, Kurdistan University of Medical Sciences, Sanandaj, IR Iran

**Keywords:** Congenital Hypothyroidism, Propensity Score, Sex Characteristics, Interaction, Iran

## Abstract

**Background:**

Several studies have demonstrated an increased risk of congenital hypothyroidism (CH) in girls in comparison to boys.

**Objectives:**

The aim of this study was to determine the potential interactions that are able to change the effect of gender on congenital hypothyroidism.

**Patients and Methods:**

We conducted a matched 1:4 case-control study in Hamadan Province, western of Iran, from 2005 to 2011. Based on screening program data, neonates with TSH ≥ 10 mU/L and T4 ≤ 6.4 μg/dL were considered as congenital hypothyroidism (Cases). Cases and controls were matched regarding the year and place of birth. Data was analyzed using two different approaches including propensity score and multiple conditional logistic regression model.

**Results:**

A total of 277 cases and 1036 controls were included in the study. Girls accounted for 57.4% of the cases and 51.2% of controls (P = 0.065). Based on the multiple conditional logistic regressions, only the interaction of gender (girl) and birth season (summer) increased the likelihood of CH significantly (OR = 3.09; 95% CI: 1.09-8.74; P = 0.034). On the other hand, the ORs of the interaction of gender and all other factors (except for birth season) were not statistically significant in CH.

**Conclusions:**

Birth season might act as an interaction that is able to increase the risk of CH in girls. Accordingly, awareness of the birth season could help policymakers who plan preventive programs to reduce the false negative results among neonates, especially girls.

## 1. Background

Congenital hypothyroidism (CH) is one of the most common congenital endocrine disorders classified into permanent and transient CH (1). Newborn screening programs for CH is one of the most important ways of early detection and treatment of the disorder as well as providing the opportunity to investigate the CH etiology and pathogenesis (2). In Iran, the pilot of screening program for CH was first established in three provinces in 1997 and then progressively developed all over the country in 2005 (3). According to the reports of CH screening, its incidence is higher in Iran (1 – 2.7 in 1,000 live births) (4-6) in comparison to the global average of CH, which is one per 3000 to 4000 of live births (1, 7).

Numerous epidemiological studies were conducted to investigate the risk factors of CH. Many studies reported that CH was associated with individual and environmental factors, including gender (8), birth weight (9), race and ethnicity (10), mother’s age (8), gestational age (8, 11), consanguinity (12), parental education (13), type of labor (14), birth weight (15), twin (11), birth season (16, 17), and drug usage during pregnancy (1). All of these studies were limited to identify the CH risk factors to conduct new prevention strategies.

Hormones are the basis of many biological differences between both sexes (18). According to previous evidence, increased risk of CH had been reported in girls in comparison to boys (8, 10, 11, 19). In addition, based on the etiology of CH, most studies reported considerably higher risks of both athyreosis and ectopia in girls in contrast to boys (15, 20, 21). However, it is still unclear why females are more susceptible to develop CH. Therefore, there is a need to conduct a study to determine the risk factors of CH by gender subgroups.

## 2. Objectives

To our knowledge, this is the first study to assess CH risk factors among Iranian neonates by gender groups. We investigated the interaction between gender and other investigated factors to CH occurrence in neonates.

## 3. Patients and Methods

This matched case-control study was part of an MSc thesis, approved by the local Human Subject Review Board of Hamadan University of Medical Sciences and conducted in Hamadan Province, western of Iran, in 2012. In Iran, neonatal CH screening program is primarily based on TSH measurement in filter paper blood spots, All of the neonates born in Hamadan Province between September 2005 and March 2011 were screened for CH between three to five days of age. Those with suspicious test results (TSH > 5 mU/L based on capillary blood collected from a heel stick and adsorbed onto filter paper) were tested for TSH and T4 and finally, the diagnosis of CH was confirmed by serum TSH ≥ 10 mU/L and T4 ≤ 6.4 μg/dL (Cases) (3). Sample size was calculated based on the census method. Neonates who did not develop CH (Controls) with regard to the normal TSH and T4 titers were considered as controls. Based on year and place of birth. Cases were matched at a 1:4 ratio with controls. Details were previously explained (22).

In our study, we investigated only the effect of the potential risk factors with a significant association with CH in an unadjusted regression model (22). Variables including the neonates’ sex, weight, jaundice at birth (in the first 24 hours after birth), twin, father’s educational level, smoking status, parents’ consanguinity, gestational age, mother’s age, type of delivery (vaginal discharge, emergency cesarean, or elective cesarean), term or preterm (before 37 weeks of gestation) delivery, birth season, maternal anemia (hemoglobin concentration < 11 g/dL during pregnancy (23)), and maternal goiter (goiter during pregnancy) were extracted from the univariate regression model.

To consolidate the strength of our findings, we used two statistical methods to estimate the risk associated with CH. Firstly, we performed a propensity score matched logistic regression model. In this approach, we adjusted the models for other factors for minimizing the effect of other potential confounding factors in assessing each variable. We individually matched each case to one control based on the propensity score. Secondly, we constructed conditional logistic regression models to derive both unadjusted and adjusted odds ratios (OR) and 95% confidence intervals (95% CI) as well as interaction term to check any interaction between the two factors. In our analysis, one of the two factors was gender, which was checked with other factors one by one.

Data processing and analysis were performed at 0.05 significance level using the statistical software Stata 11 (StataCorp, College Station, TX, USA).

## 4. Results

A total of 277 cases and 1036 controls were included in the study. Girls accounted for 57.4% of cases and 51.2% of controls (female-male ratio: 1.2:1, P = 0.065). The neonates' mean birth weight were 3040.2 ± 725.5 gr and 2952.8 ± 704.2 gr in girls and boys, respectively (P = 0.027). Table 1 summarizes unadjusted and adjusted odds ratios and their corresponding 95% CIs as well as interaction term.

According to univariate conditional logistic regression results, there were statistically significant associations between the risk of CH and the investigated factors including birth season (winter), prematurity, father’s education and smoking, maternal goiter and anemia, parents’ consanguinity, mother’s age, jaundice at birth, and the type of delivery. The same results were found with a series of fluctuation (strong, dilute, or no change) in multiple conditional logistic regression when the gender variable was added to the model.

The interactions were analyzed by using the multiple conditional logistic regressions including the interaction terms between independent variables and gender on CH occurrence. Based on this model, only the interaction of gender (girl) and birth season (summer) significantly increased the likelihood of CH (OR = 3.09; 95% CI: 1.09-8.74; P = 0.034). On the other hand, the ORs of the interaction of gender and all other factors on CH were not statistically significant. According to the results of both univariate and multiple conditional logistic regressions, there was no association between CH and gestational age, birth weight, and twin. 

**Table 1.** Analysis of Univariate and Multivariate Conditional Logistic Regression as Well as Interaction Term

Variables	Crude OR ^a^ (95% CI)	Adjusted ^b^ OR (95% CI)	Interaction Term With Gender (Reference Category: Boy)^c^	P Value
**Twin**				
No	1.00	1.00	1.00	
Yes	1.00 (0.61, 1.63)	1.01 (0.62, 1.66)	0.66 (0.21, 1.51)	0.255
**Birth season**				
Spring	1.00	1.00	1.00	
Summer	1.31 (0.79, 2.17)	1.29 (0.78, 2.13)	3.09 (1.09, 8.74) ^d^	0.034
Fall	0.81 (0.49, 1.36)	0.81 (0.49, 1.36)	1.62 (0.58, 4.53)	0.354
Winter	2.23 (1.37, 3.64) ^d^	2.23 (1.37, 3.64) ^d^	1.24 (0.47, 3.31)	0.659
**Birth situation**				
Term	1.00	1.00	1.00	
Preterm ^e^	2.71 (1.65, 4.47) ^d^	2.75 (1.67, 4.54) ^d^	1.46 (0.53, 4.00)	0.464
**Jaundice at birth ** ^**f**^				
No	1.00	1.00	1.00	
Yes	3.98 (2.71, 5.85) ^d^	4.01 (2.72, 5.90) ^d^	0.85 (0.39, 1.84)	0.676
**Birth weight, g**				
2500-3500	1.00	1.00	1.00	
< 2500	0.88 (0.58, 1.33)	0.88 (0.59, 1.34)	0.89 (0.39, 2.05)	0.791
> 3500	0.65 (0.41, 1.01)	0.65 (0.41, 1.02)	1.26 (0.51, 3.15)	0.615
**Maternal age, y**				
18-35	1.00	1.00	1.00	
36-43	0.55 (0.36, 0.82) ^d^	0.56 (0.36, 0.86) ^d^	1.20 (0.50, 2.88)	0.677
**Maternal anemia**				
No	1.00	1.00	1.00	
Yes	2.23 (1.37, 3.62) ^d^	2.28 (1.40, 3.70) ^d^	1.04 (0.39, 2.75)	0.937
**Maternal goiter history ** ^**g**^				
No	1.00	1.00	1.00	
Yes	2.85 (1.68, 4.85) ^d^	2.84(1.67, 4.83) ^d^	1.11 (0.38, 3.24)	0.852
**Father’s education**				
Academic	1.00	1.00	1.00	
Non academic	1.72 (1.11, 2.68) ^d^	1.70 (1.09, 2.64) ^d^	1.89 (0.77, 4.60)	0.163
**Father’s smoking**				
No	1.00	1.00	1.00	
Yes	1.78 (1.13, 2.82) ^d^	1.78 (1.12, 2.81) ^d^	1.43 (0.57, 3.59)	0.445
**Gestational age, wk**				
37 - 40	1.00	1.00	1.00	
< 37	1.17 (0.72, 1.89)	1.20 (0.73, 1.95)	0.76 (0.29, 2.03)	0.588
> 40	1.36 (0.91, 2.05)	1.38 (0.92, 2.10)	0.68 (0.30, 1.54)	0.356
**Delivery type**				
Vaginal discharge	1.00	1.00	1.00	
Emergency CS	3.11 (2.06, 4.70) ^a^	3.13 (2.07, 4.74) ^a^	1.19 (0.52, 2.74)	0.677
Elective CS	6.57 (3.65, 11.83) ^a^	6.49 (3.60, 11.71) ^a^	1.01 (0.31, 3.33)	0.986
**Parents’ consanguinity**				
No relation	1.00	1.00	1.00	
First cousin	1.79 (1.14, 2.82) ^a^	1.77 (1.12, 2.78) ^a^	0.72(0.28, 1.80)	0.478
Second cousin	7.01 (4.32, 11.36) ^a^	6.98 (4.30, 11.32) ^a^	2.02 (0.76, 5.39)	0.160

^a^ Abbreviations: CI, confidence interval; CS, cesarean; OR, odds ratio.

^b^ Adjusted for the gender effect.

^c^ Gender × Related Factor.

^d^ P < 0.05.

^e^ preterm: before 37 weeks of gestation.

^f^ Jaundice at birth: in the first 24 hours after birth.

^g^ Maternal goiter history: goiter during pregnancy.

## 5. Discussion

The main finding of the present study was that birth season (summer) accompanied with girl gender increased the risk of CH although any of these factors had little effect on the risk of CH individually. Based on the univariate conditional logistic regression, our result are in agreement with Ordookhani et al. (24) findings which showed that CH occurred significantly more in winter than other seasons. The incidence of CH was shown to vary seasonally in a number of studies in different parts of world including Iran (16, 17), Japan (25) and the West Midlands of England (26). Gu et al. (25) found sex-specific seasonal patterns of CH incidence in Japan. They reported that from January to December, males had one peak, while females had two peaks. In addition, Hashemipour et al. (16) showed a different pattern of birth month among neonates in Isfahan. They reported that there were a peak and nadir incidence of CH in summer and in the last month of autumn, respectively. They also concluded that exposure to chemical compounds, seasonal environmental factors, and differences in climate might play role in the etiology of CH. However, seasonality was not demonstrated in a number of other studies including those in the North of England (27), Italy (28), and Canada (29).

Many reports have indicated that CH is frequently found in girls (10, 11, 19). Moreover, epidemiological studies have revealed that the clinical risk factors for some diseases are also different between gender groups (21, 30). However, the present study was the first epidemiological study in Iranian neonates, which determined CH risk factors by taking into account the interaction term of gender.

In a previous report, we proposed that several prognostic factors such as twin, birth season, maturity, jaundice at birth, birth weight, maternal age, maternal anemia and goiter, gestational age, delivery type, father’s education level and smoking status, and consanguinity might be the key factors underlying the relatively high incidence of CH in investigated neonates (22). Although the female-to-male ratio was 1.2:1 among neonates, there was no significant difference in CH prevalence with regard to gender. According to previous evidences, females to males ratio was approximately 1.0 among hereditary cases of CH; moreover, this ratio was about 2.0 for the CH cases with both athyreosis and ectopia groups (20). Castanet et al. (31) reported that the female preponderance over males for isolated CH was similar for patients with athyreosis or an ectopic thyroid gland. It means that the preponderance of female cases is mostly associated with dysgenesis of the thyroid gland. These results were also reported in another study (15).

Results of the present study indicated that odds ratio estimates of CH for investigated factors (except for birth season) did not differ substantially between girls and boys. This is in line with other researchers findings. Ng et al. found that there was no significant difference between girls and boys regarding gestation and birth weight in all etiological subgroups such as athyreosis and ectopia groups (20). On the other hand, Van Vliet et al. showed that boys with CH had a higher incidence of absent knee epiphyses at diagnosis in comparison to girls and this sexual dimorphism was seen in all gestational ages (15).

It is unclear why girls have a higher incidence rate of CH than boys, while there is no difference in proportion of other risk factors between them. We do not know the underlying causes of this difference exactly. This study had some strengths and limitations. A main strength of this study was that cases were selected based on census method between 2005 and 2011, and four controls were individually matched for each case. To consolidate the strength of our findings we used two statistical adjusted methods to estimate the ORs as well. The primary limitation of the study was its small sample size in some subgroups. Moreover, we performed two different multivariate analyses to decrease the confounding, but residual confounding was still inevitable.

Our findings indicated that birth season might act as an interaction to increase the risk of CH in girls. Accordingly, awareness of the birth season could help policymakers who plan preventive programs to reduce the false negative results among neonates, especially girls. Whilst the CH prevalence was higher among girls than boys, but its reasons deserve further investigations to be elucidated. These observations need to be validated in larger epidemiological studies.
